# Effect of PCSK9 inhibitors on pulse wave velocity and monocyte-to-HDL-cholesterol ratio in familial hypercholesterolemia subjects: results from a single-lipid-unit real-life setting

**DOI:** 10.1007/s00592-021-01703-z

**Published:** 2021-03-21

**Authors:** Roberto Scicali, Antonino Di Pino, Viviana Ferrara, Agata Maria Rabuazzo, Francesco Purrello, Salvatore Piro

**Affiliations:** Department of Clinical and Experimental Medicine, University of Catania, Internal Medicine, Garibaldi Hospital, Via Palermo 636, 95122 Catania, Italy

**Keywords:** Familial hypercholesterolemia, PCSK9 inhibitors, Pulse wave velocity, Innate immunity, Inflammatory profile, Cardiovascular risk

## Abstract

**Aims:**

Subjects with familial hypercholesterolemia (FH) are characterized by an increased amount of low-density lipoprotein cholesterol (LDL-C) that promotes a continuous inflammatory stimulus. Our aim was to evaluate the effect of PCSK9-i on inflammatory biomarkers, neutrophil-to-lymphocyte ratio, monocyte-to-high-density lipoprotein ratio (MHR), and on early atherosclerosis damage analyzed by pulse wave velocity (PWV) in a cohort of FH subjects.

**Methods:**

In this prospective observational study, we evaluated 56 FH subjects on high-intensity statins plus ezetimibe and with an off-target LDL-C. All subjects were placed on PCSK9-i therapy and obtained biochemical analysis as well as PWV evaluation at baseline and after six months of PCSK9-i therapy.

**Results:**

After six months of add-on PCSK9-i therapy, only 42.9% of FH subjects attained LDL-C targets. As expected, a significant reduction of LDL-C (− 49.61%, *p* < 0.001) was observed after PCSK9-i therapy. Neutrophil count (NC) and MHR were reduced by PCSK9-i (-13.82% and -10.47%, respectively, *p* value for both < 0.05) and PWV significantly decreased after PCSK9-i therapy (− 20.4%, *p* < 0.05). Finally, simple regression analyses showed that ∆ PWV was significantly associated with ∆ LDL-C (*p* < 0.01), ∆ NC and ∆ MHR (*p* value for both < 0.05).

**Conclusions:**

In conclusion, PCSK9-i therapy significantly improved lipid and inflammatory profiles and PWV values in FH subjects; our results support the positive effect of PCSK9-i in clinical practice.

## Introduction

Atherosclerosis is a long-term damaging process and its progression leads to cardiovascular system injury [1]. Several environmental and genetic factors play a part in atherosclerosis progression; among these, an increased plasma level of low-density lipoprotein LDL cholesterol (LDL-C) is causatively associated with atherosclerotic cardiovascular disease (ASCVD) [2]. However, the full definition of atherosclerosis as a chronic deposition of LDL-C may be not exhaustive; in fact, other processes are involved in the pathogenesis and progression of atherosclerosis together with LDL cholesterol.

In the last few years, several studies have indicated the importance of an inflammatory state in the pathophysiology of ASCVD [3, 4]; in particular, inflammation appears to be the final expression of the systemic interaction between hypercholesterolemia and the immune system during atherosclerosis progression [5]. In subjects with hypercholesterolemia, previous studies showed that lipid storage in the arterial wall promoted the inflammatory cascade and thus the migration of immune cells such as monocyte-derived macrophages and T lymphocyte subtypes into the inflammatory lipid-wall injury [6, 7]. The concept of atherosclerosis as a continuous inflammatory process promoted by a persistent LDL-C level and immune system activation may explain why, despite lifestyle changes and lipid-lowering treatments (LLT), ASCVD is still considered the leading cause of disease burden and death [8]. Increasing attention has been recently focused on novel inflammatory biomarkers largely available in clinical practice such as neutrophil-to-lymphocyte ratio (NLR) and monocyte-to-high-density lipoprotein ratio (MHR); in fact, several studies showed that these inflammatory biomarkers satisfactorily predicted ASCVD in the general population [9, 10]. In this context, NLR and MHR may be useful to better discriminate the “athero-inflammatory” profile in the general population and, in particular, in subjects with high LDL-C levels such as familial hypercholesterolemia (FH) [11].

FH is the most frequent monogenic genetic disorder characterized by an increased LDL-C level from childhood [12]. If not treated, FH subjects experience both an increase of pulse wave velocity (PWV) and subsequently premature ASCVD [13]; thus, early diagnosis and LLT are needed to ameliorate the cardiovascular prevention in FH subjects [14]. However, despite intensive statin therapy, few FH subjects obtain the recommended lipid targets, while ASCVD affects the majority of FH subjects [15, 16].

In the era of novel LLT, more attention has been paid to the inhibitors of proprotein convertase subtilisin/kexin type 9 (PCSK9-i) [17]. PCSK9-i leads to a reduction of circulating PCSK9 and thus reduces LDL receptor cleavage and enhances its presence on the liver cell surface [18]. The clinical efficacy of PCSK9-i was shown in previous studies [19, 20]; in particular, the reduction of LDL-C and ASCVD by PCSK9-i was ≃50–60% and 15%, respectively. Because of its cardiovascular properties, PCSK9-i therapy is a crucial LLT in subjects at high cardiovascular risk such as FH [21]. No data exist regarding the effect of PCSK9-i on NLR and MHR in these subjects.

In this study, we aimed to evaluate the effect of PCSK9-i on the inflammatory biomarkers NLR and MHR and on early atherosclerosis damage evaluated by pulse wave velocity (PWV) in a cohort of FH subjects.

## Methods

### Study design and population

This was an open-label, prospective, observational study involving subjects with a previously confirmed FH genetic diagnosis [22]. All participants were enrolled from the Lipid Centre of the Garibaldi Hospital/University of Catania, Sicily, Italy, from October 2018 to September 2020. All participants were aged between 18 and 70 years and were on high-intensity statins (atorvastatin 40–80 mg, rosuvastatin 20–40 mg) plus ezetimibe for at least six months at the time of enrollment. Finally, all participants had an LDL-C beyond the recommended targets according to the European Society of Cardiology/European Atherosclerosis Society guidelines 2019 for the management of dyslipidemias. After a 12-h fast, all participants had a hematological and clinical evaluation. For all participants, biochemical analyses and PWV evaluations were performed at baseline (T0) and after six months (T1) of PCSK9-i therapy. Body weight and height were measured, and body mass index (BMI) was calculated as weight divided by the squared value of height (kg/m2) [23]. Arterial hypertension was defined as brachial blood pressure (BP) ≥ 140 mm Hg (systolic) and/or 90 mm Hg (diastolic) on at least two different occasions, or if the subjects were on antihypertensive therapy [23]. Statin therapy was defined as a daily intake of statins; duration of statin therapy was defined as the numbers of years on statin therapy [24]. Non-statin lipid-lowering therapy was defined as the intake of lipid-lowering treatments other than statins (niacin, bile acid sequestrants, fibrates, nutraceuticals) [24]. Type 2 diabetes (T2D) was defined as a fasting plasma glucose ≥ 126 mg/dL on two consecutive readings or a glycated hemoglobin (HbA1c) ≥ 6.5% or the use of anti-diabetic medications [25]. Smokers were divided into either current smoker (defined as any cigarette in the last month) or past smoker [26]. ASCVD was defined as documented previous myocardial infarction, acute coronary syndrome, coronary revascularization (percutaneous coronary intervention or coronary artery bypass graft surgery) or other arterial revascularization procedures, stroke or transient ischemic attack, or atherosclerotic artery disease [27]. LDL-C target was defined as an LDL-C < 70 mg/dl for FH subjects without ASCVD or an LDL-C < 55 mg/dl for FH subjects with ASCVD [28]. Exclusion criterion was the use of non-statin lipid-lowering therapy.

### Biochemical analysis

Fasting plasma glucose (FPG) as well as serum total cholesterol (TC), TG, high-density lipoprotein cholesterol (HDL-C), high-sensitivity C-reactive protein (hs-CRP), aspartate transaminase (AST), alanine transaminase (ALT) and creatine phosphokinase (CPK) were assessed by available enzymatic methods [29]. Apolipoprotein B (ApoB) and apolipoprotein A1 (ApoA1) were evaluated with a nephelometer assay (Siemens AG Healthcare Sector, Erlangen, Germany) [30]. Levels of lipoprotein (a) [Lp(a)] were measured with the latex agglutination immunoassay [31]. LDL-C was obtained by the Friedewald formula. Non-HDL cholesterol (non-HDL-C) was derived from baseline values. HbA1c was measured with high-performance liquid chromatography using a National Glycohemoglobin Standardization Program and standardized to the Diabetes Control and Complications Trial assay [32]. Chromatography was performed using a certified automated analyzer (HPLC; HLC-723G7 hemoglobin HPLC analyzer; Tosoh Corp.; normal range 4.25–5.9% [23–41 mmol/mol]). White blood cell count was performed by a blood cell analyzer (UniCel DxH-900, Beckman Coulter, Milan, Italy). NLR and MHR were derived from baseline values.

### Pulse wave velocity evaluation

The SphygmoCor CVMS (AtCor Medical, Sydney, Australia) system was used for PWV evaluation. This system is made up of a tonometer and 2 different pressure waves recorded at the common carotid artery (proximal recording site) and at the femoral artery (distal recording site). The distance between the recording sites and the suprasternal notch was measured by a tape measure. An electrocardiogram was performed to determine the start of the pulse wave. The PWV was defined as the difference in the time interval of the pulse wave between the 2 different recording sites and the heart, divided by the travel distance of the pulse waveform. The PWV was calculated on the mean of 10 consecutive pressure waveforms to perform a complete respiratory cycle [33].

### Statistical analysis

The distributional characteristics of each variable, including normality, were assessed by the Kolmogorov–Smirnov test. Data are reported as mean ± standard deviation (SD) for continuous parametric parameters and median (interquartile range-IQR) for continuous nonparametric variables and as frequency (percentage) for categorical variables. When necessary, continuous nonparametric variables (TG, Lpa, CPK, duration of statin therapy and hs-CRP) were logarithmically transformed to reduce skewness. The χ2 test was used for categorical variables. The changes of TC, HDL-C, TG, LDL-C, non-HDL-C, ApoB, ApoAI, ApoB/ApoAI, Lpa, FPG, HbA1c, BMI, systolic BP, diastolic BP, hs-CRP, CPK, GOT, GPT, NLR, MHR and PWV, from baseline (T0) for all the treatment time (T1 vs T0), were evaluated as delta (∆) and calculated according to the following formula: [(T1-T0)/T0)*100]. To test time-dependent differences (T1 vs T0) of clinical and biochemical characteristics in the study population, we used Student’s t test. Finally, simple regression analyses were performed to relate ∆ PWV with ∆ LDL-C, ∆ NLR and ∆ MHR. All statistical analyses were performed using IBM SPSS Statistics package for Windows version 23. For all tests, p < 0.05 was considered significant.

The study was approved by the local ethics committee (prot. Number 46/19) in accordance with the ethical standards of the institutional and national research committees and with the 1964 Declaration of Helsinki and its later amendments or comparable ethical standards. Informed consent was obtained from each subject enrolled in the study.

## Results

**Fig. 1:**
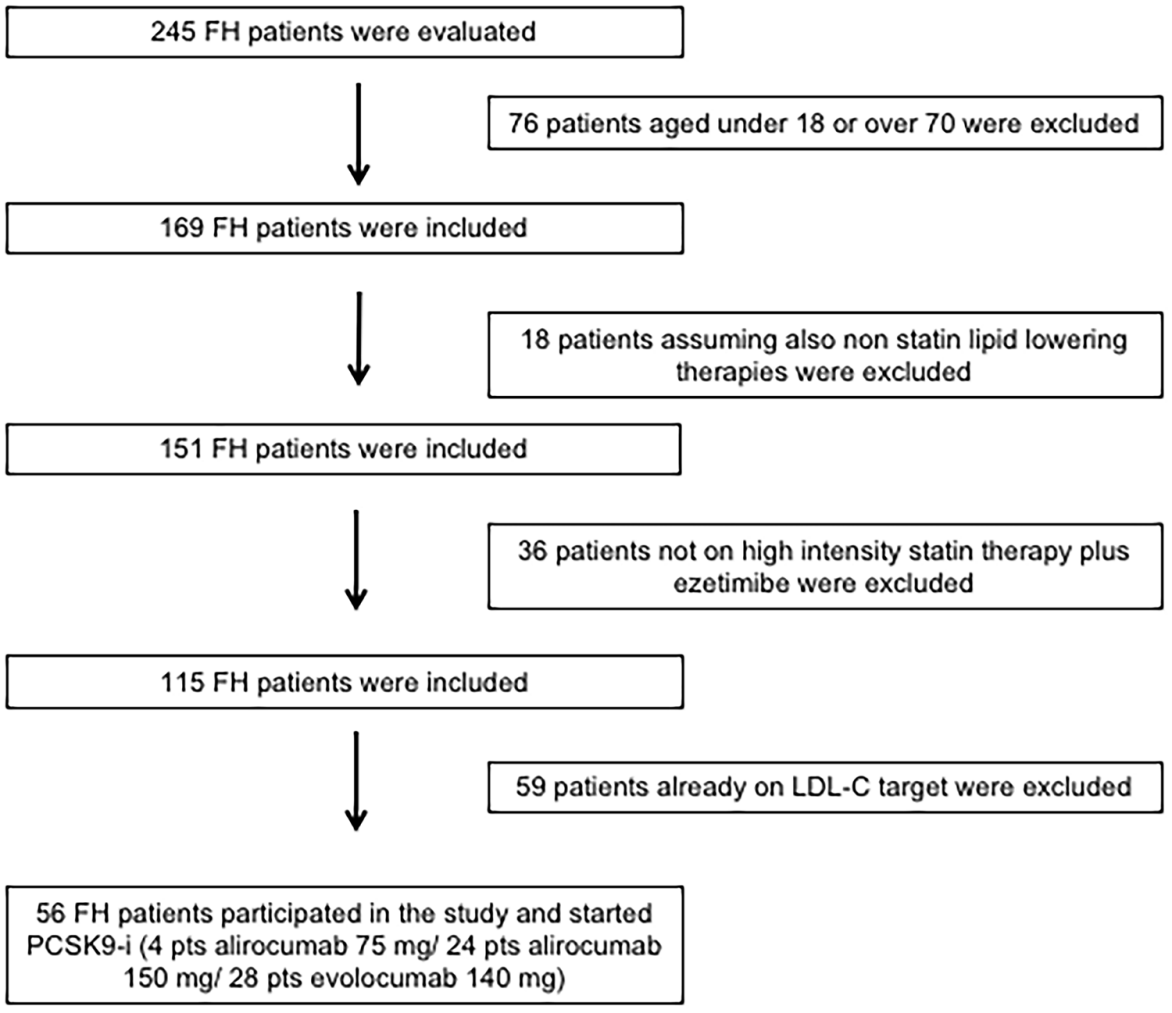
Enrollment flowchart of the study population. FH = familial hypercholesterolemia, PCSK9-i = proprotein convertase subtilisin/kexin type 9 inhibitors

In total, 245 FH subjects were evaluated; of these, 56 subjects satisfied the inclusion criteria and participated in this open-label, prospective, observational study. According to the lipid-lowering recommendation of the European Society of Cardiology/European Atherosclerosis Society guidelines 2019 for the management of dyslipidemias and the Italian reimbursement rules of PCSK9-i, all FH subjects started PCSK9-i therapy; in particular, 4 subjects added alirocumab 75 mg, 24 subjects added alirocumab 150 mg, and 28 subjects added evolocumab 140 mg (Fig. 1).

Table 1 shows the characteristics of the study population; 46.4% of FH subjects had a prior ASCVD. All subjects were heterozygous FH, and the majority of subjects had an LDLR genetic variant; furthermore, more subjects were on rosuvastatin 20 mg than atorvastatin 40 mg and 39.3% of subjects were on antihypertensive therapy.

**Table 1:** Characteristics of the study population. Data are presented as mean ± standard deviation, percentages, or median (interquartile range). FH = familial hypercholesterolemia, ASCVD = atherosclerotic cardiovascular disease, LDLR = low-density lipoprotein receptor, ApoB = apolipoprotein B

*Demographic characteristics*	
N	56
Age, years	56.38 ± 6.89
Men, *n*(%)	28 (50)
Smokers, *n*(%)	12 (21.4)
ASCVD, *n*(%)	26 (46.4)
*FH genotype*	
LDLR, *n*(%)	
55 (98.2)	
ApoB, *n*(%)	1 (1.8)
*Mutation class*	
Amino acid change, *n*(%)	
30 (53.6)	
Null allele, *n*(%)	26 (46.4)
*FH phenotype*	
Heterozygous FH, *n*(%)	56 (100)
*Treatment*	
Duration of statin therapy, years	
12 (2–20)	
Duration of ezetimibe therapy, years	5 (1–7)
Antihypertensive therapy, *n*(%)	22 (39.3)
*High-intensity statin therapy*	
Atorvastatin 40 mg, *n*(%)	22 (39.3)
Rosuvastatin 20 mg, *n*(%)	34 (60.7)

After six months of add-on PCSK9-i therapy, only 42.9% of FH subjects attained LDL-C targets according to the European Society of Cardiology/European Atherosclerosis Society guidelines 2019 for the management of dyslipidemias. As expected, PCSK9-i significantly reduced TC, LDL-C, non-HDL-C and ApoB plasma levels (− 35.35%, − 49.61%, − 44.16% and − 43.36%, respectively), whereas no difference was found on TG, HDL-C, ApoAI and Lp (a). Furthermore, glucose profile was unvaried after adding-on PCSK9-i therapy; at baseline 3 FH subjects were diabetics and no new cases of T2D were detected after adding-on PCSK9-i therapy. BMI, as well as systolic and diastolic BP, was unchanged after adding-on PCSK9-i therapy; moreover, liver and muscle enzymes were similar before and after PCSK9-i therapy (Table 2).

**Table 2:** Glucose, lipid, risk factor, liver and muscle enzyme profiles of the study population at baseline and after six months of add-on PCSK9-i therapy

	FH subjects (n = 56)	FH subjects (n = 56)	∆	*p* Value between the two groups
	Baseline	6-month add-on PCSK9-i		
*Glucose profile*				
FPG, mg/dL	93.86 ± 9,31	92.59 ± 9,26	− 1.35%	0.67
HbA1c, %	5.62 ± 0.47	5.74 ± 0.4	2.14%	0.24
Type 2 Diabetes, n (%)	3	3	–	–
*Lipid profile*				
TC, mg/dL	221.81 ± 19.48	143.39 ± 18.35	− 35.35%	< 0.001
HDL, mg/dL	52.3 ± 9.65	54.45 ± 9.22	4.11%	0.45
TG, mg/dL	98.5 (63.5–120.25)	90.5 (61.25–111.5)	− 8.12%	0.27
LDL-C, mg/dL	148.47 ± 16.88	74.81 ± 16.33	− 49.61%	< 0.001
LDL-C target, n (%)	–	24 (42.9)	–	–
Non-HDL-C, mg/dL	169.12 ± 16.66	94.43 ± 17.37	− 44.16%	< 0.001
ApoB, mg/dL	109.36 ± 14.95	61.94 ± 13.99	− 43.36%	< 0.001
ApoAI, m g/dL	127.43 ± 13.28	129.76 ± 12.86	1.83%	0.56
ApoB to ApoAI ratio	0.87 ± 0.25	0.48 ± 0.19	− 44.82%	< 0.001
Lp(a), nmol/L	47.5 (21.1–66.8)	35.2 (11.5–48.7)	− 25.89%	0.34
*Risk factors*				
Body mass index, kg/m^2^	26.15 ± 2.13	26.1 ± 2.11	− 0.19%	0.87
Systolic BP, mmHg	120.25 ± 9.44	118.15 ± 9.91	− 1.75%	0.45
Diastolic BP, mmHg	71.5 ± 10.1	70.4 ± 10.2	− 1.54%	0.65
*Liver and muscle enzymes*				
AST, U/L	26.07 ± 8.48	25.03 ± 8.21	− 3.99%	0.76
ALT, U/L	29.76 ± 9.79	28.53 ± 9.51	− 4.15%	0.71
CPK, U/L	113.5 (82.0–153.5)	121.5 (88–157.25)	7.05%	0.61

Data are presented as mean ± SD, percentages, or median (interquartile range). *PCSK9-I* proprotein convertase subtilisin/kexin type 9 inhibitors, *FH *familial hypercholesterolemia, FPG = fasting plasma glucose, HbA1c = glycated hemoglobin, *TC* total cholesterol, *HDL* high-density lipoprotein, *TG* triglycerides, *LDL-C* low-density lipoprotein cholesterol, *TG/HDL *triglyceride to high-density lipoprotein ratio, *ApoB* apolipoprotein B, *ApoAI* apolipoprotein AI, *Lp*(a) lipoprotein (a), *BP *blood pressure, *AST *aspartate transaminase, *ALT *alanine transaminase, *CPK *creatine phosphokinase

Table 3 shows the white blood cell count and inflammatory profiles after six months of PCSK9-i therapy. PCSK9-i therapy significantly reduced the neutrophil count (NC) (5.21 ± 1.04 vs 4.49 ± 0.95 × 10^3^/µL [∆ − 13.82%], *p* < 0.05), while the monocyte count was reduced without reaching statistical significance; furthermore, the lymphocyte count was unchanged after six months of PCSK9-i therapy. As concerns the inflammatory profile, PCSK9-i therapy significantly reduced MHR (11.56 ± 4.14 vs 10.35 ± 4.16 [∆ − 10.47%], *p* < 0.05), while hs-CRP values were similar before and after PCSK9-i therapy.

**Table 3:** White blood cell count and inflammatory profiles of the study population at baseline and after six months of add-on PCSK9-i therapy

	FH subjects (*n* = 56)	FH subjects (*n* = 56)	∆	*p* Value between the two groups
	Baseline	6-month add-on PCSK9-i		
*White blood cell count*				
WBCC, 10^3^/µL	8.14 ± 1.16	7.18 ± 1.26	− 11.79%	0.07
NC, 10^3^/µL	5.21 ± 1.04	4.49 ± 0.95	− 13.82%	< 0.05
LC, 10^3^/µL	2.14 ± 0.47	2.11 ± 0.76	− 0.01%	0.67
MC, 10^3^/µL	0.62 ± 0.17	0.55 ± 0.16	− 11.29%	0.08
*Inflammatory profile*				
hs-CRP, mg/dL	0.19 (0.08–0.35)	0.20 (0.09–0.37)	5.26%	0.87
NLR	2.6 ± 0.72	2.5 ± 0.7	− 3.85%	0.45
MHR	11.56 ± 4.14	10.35 ± 4.16	− 10.47%	< 0.05

Data are presented as mean ± SD, percentages, or median (interquartile range). PCSK9-i = proprotein convertase subtilisin/kexin type 9 inhibitors, *FH *familial hypercholesterolemia, *WBCC *white blood cell count, *NC *neutrophil count, *LC *lymphocyte count, *MC *monocyte count, *hs-CRP *high-sensitivity C-reactive protein, NLR = neutrophil-to-lymphocyte ratio, MHR = monocyte-to-high-density lipoprotein ratio

**Fig. 2:**
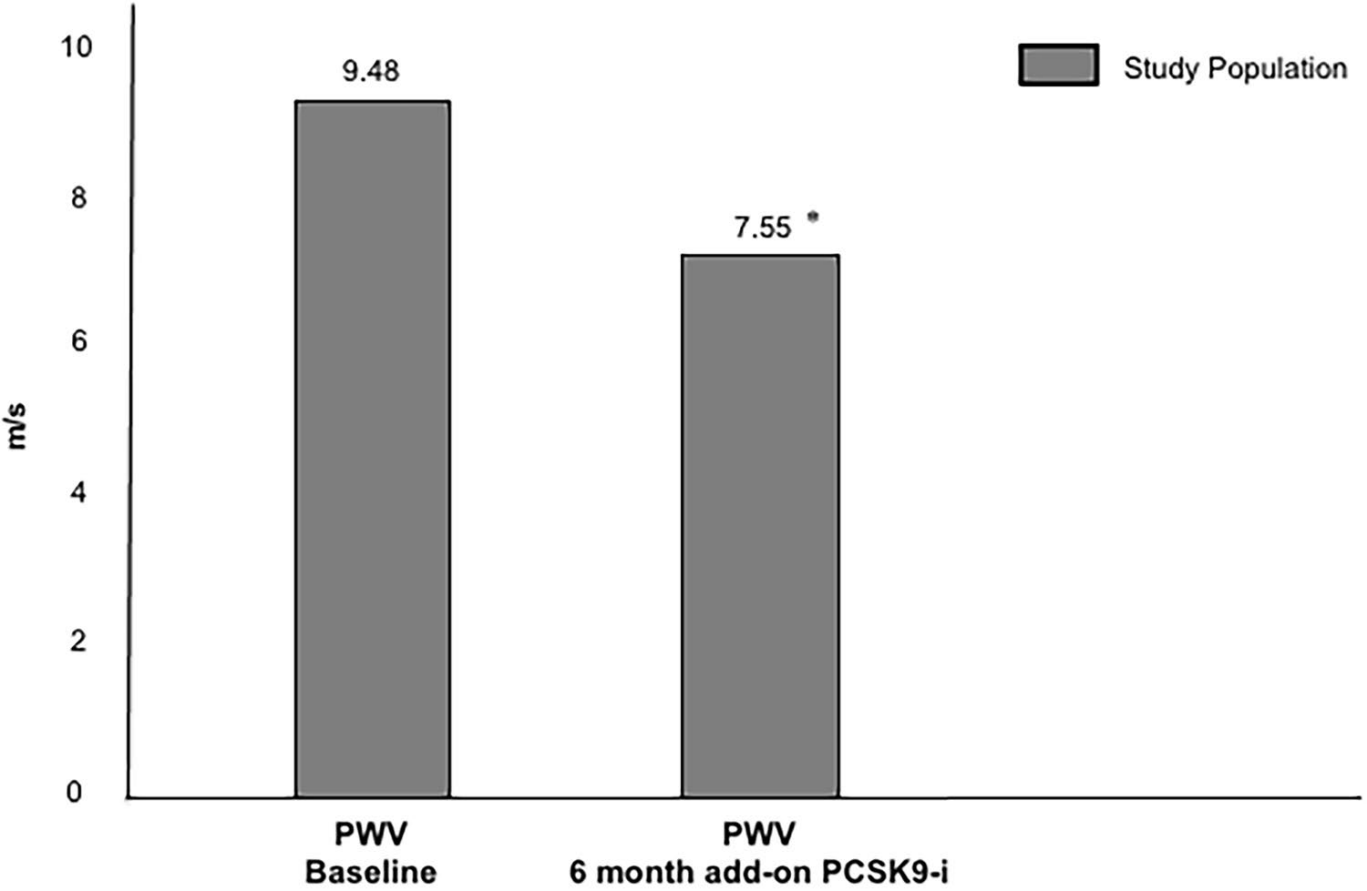
PWV values of the study population after six months of add-on PCSK9-i. PWV = pulse wave velocity, PCSK9-i = proprotein convertase subtilisin/kexin type 9 inhibitors. *P value < 0.01 vs baseline

As illustrated in Fig. 2, PWV significantly ameliorated after six months of PCSK9-i therapy (9.48 ± 1.45 vs 7.55 ± 1.43 m/s [∆ − 20.4%], *p* < 0.01).

Finally, simple regression analyses showed that ∆ PWV was significantly associated with ∆ LDL-C (*p* < 0.01), ∆ NC and ∆ MHR (*p* value for both < 0.05) (Table 4).

**Table 4:** Simple linear regression analyses evaluating ∆ PWV as a dependent variable

Dependent variable	∆ PWV	
Independent variable	Coefficient β	*P* value
∆ LDL-C, %	1.635 ± 0.165	< 0.01
∆ NC, %	1.249 ± 0.110	< 0.05
∆ MHR, %	1.283 ± 0.112	< 0.05

*∆ PWV *change of pulse wave velocity from baseline for all the PCSK9-i therapy duration, *∆ LDL-C *change of low-density lipoprotein cholesterol from baseline for all the PCSK9-i therapy duration, *∆ NC *change of neutrophil count from baseline for all the PCSK9-i therapy duration, *∆ MHR *change of monocyte-to-high-density lipoprotein ratio from baseline for all the PCSK9-i therapy duration

## Discussion

Over the last few years, increasing attention has been given to the synergic role of LDL-C and inflammatory status in atherosclerotic injury; the coexistence of these atherogenic conditions may be crucial in subjects at high cardiovascular risk such as FH subjects. In these subjects, LDL-C appears to be the main trigger of inflammatory status that promotes the migration of innate immune cells such as monocytes and neutrophils to the atherosclerotic lesion [34, 35]; in line with these considerations, previous studies showed that FH subjects were more inflamed than non-FH subjects and statin therapy was not able to reduce this difference [13, 36]. In this context, novel lipid-lowering strategies such as PCSK9-i may be useful to reduce LDL-C and inflammatory status in FH subjects.

In our study, we analyzed the effect of PCSK9-i on lipid profile, white blood cell count, inflammatory status and pulse wave velocity in FH subjects; to the best of our knowledge, this is the first study evaluating the role of PCSK9-i on this atherogenic axis in this population. We found that LDL-C, NC, MHR and PWV were significantly reduced after six months of adding-on PCSK9-i therapy; furthermore, simple regression analyses showed that ∆ PWV was significantly associated with ∆ LDL-C, ∆ NC and ∆ MHR.

Our findings may be related to the putative role of PCSK9 as an immune mediator in the atherosclerotic process [37]; in fact, by increasing the vascular endothelial cell expression of lectin-like oxidized low-density lipoprotein receptor-1 (LOX-1), PCSK9 plasma levels activate an inflammatory cascade promoting the migration of neutrophils and monocytes in the atherosclerotic lesion [38]. In line with these findings, Li et al. previously showed that PCSK9 plasma levels were positively associated with white blood cell count and its subtypes in subjects with coronary artery disease [39]; furthermore, Ricci et al. showed that PCSK9 promoted a pro-inflammatory stage in monocyte-derived macrophages [40]. Thus, the inhibition of PCSK9 plasma levels may diminish the risk of ASCVD by reducing LDL-C and white blood cell subtypes; in line with these considerations, in our study PCSK9-i therapy significantly reduced LDL-C and NC in FH subjects.

As regards the inflammatory profile, previous studies showed that PCSK9-i did not reduce hs-CRP levels in the general population [41] and the same finding was observed in our FH cohort; however, Kuhnast et al. showed that these drugs could suppress the inflammatory state by reducing monocyte recruitment and subsequently the necrotic core macrophages in an atherogenic mouse model [42]. In agreement with these findings, in our study we found that PCSK9-i was able to ameliorate the inflammatory state by reducing MHR in FH subjects.

PWV is a novel cardiovascular biomarker widely used in clinical practice and is a strong predictor of ASCVD in the general population [44]. It is known that statin therapy is able to reduce LDL-C as well as inflammatory profile [43, 44]; thus, these effects may better explain the statin promoted reduction of PWV in the general population [45]; thanks to these properties, statin is the first LDL-C lowering strategy in all subjects, in particular in FH subjects [46]. However, despite the use of statins, a high prevalence of premature ASCVD has been reported in FH subjects [47]. Therefore, the addition of novel lipid-lowering therapies such PCSK9-i may be helpful in reducing ASCVD risk in FH subjects. In this context, in our study we demonstrated that PCSK9-i reduced PWV and probably this effect may be the result of LDL-C and MHR reductions; in line with these considerations, in our study we found that ∆ PWV was significantly associated with ∆ LDL-C, ∆ NC and ∆ MHR.

We also found that PCSK9-i significantly reduced LDL-C by 49.61%; this was in line with previous finding by Hollstein et al. who showed a similar LDL-C decrease in a real-world setting of subjects at high ASCVD risk [48]. Furthermore, in our study a 20.4% reduction of PWV was obtained by PCSK9-i therapy and this was in line with previous studies that showed a similar effect [49, 50]. In fact, Mandraffino et al. demonstrated that PCSK9-i significantly reduced PWV compared to ezetimibe in a real-word setting of FH subjects; furthermore, Cicero et al. found a significant improvement of PWV already after three months of PCSK9-i addition.

There are several limitations to our study; first, this was an open-label, prospective, observational but not randomized study and the PCSK9-i therapeutic strategy depended on a physician’s decision. Moreover, the PCSK9-LOX1 axis was not studied. Furthermore, other parameters that may explain the interaction of LDL-C, inflammatory status and PWV such as flow mediated dilation and oxidative stress determination were not evaluated. The study population size was relatively small; however, we demonstrated a significant improvement of lipid and inflammatory profiles as well as PWV values after PCSK9-i therapy in FH subjects. These preliminary findings should be confirmed in a larger study population through defined diagnostic tools and statistical analyses. In conclusion, PCSK9-i therapy significantly ameliorated the lipid profile, inflammatory status and PWV values in a cohort of FH subjects; moreover, ∆ PWV was significantly associated with ∆ LDL-C, ∆ NC and ∆ MHR. Our findings indicate the favorable role of this novel lipid-lowering strategy on lipid and inflammatory profiles and PWV; however, a randomized controlled trial is required to evaluate the effect of PCSK9-i therapy on these pathological aspects in FH subjects.
